# Fertilitätserhaltung bei Brustkrebs – ein Risiko?

**DOI:** 10.1007/s00066-022-02037-7

**Published:** 2022-12-12

**Authors:** Kathrin Hartwig

**Affiliations:** Klinik für Strahlentherapie, Christian-Albrechts-Universität zu Kiel, Kiel, Deutschland

## Hintergrund und Ziele

Junge Frauen mit einem Mammakarzinom („breast cancer“ [BC]) erhalten oftmals eine intensivere Behandlung mit Chemotherapie. Eine häufige chemotherapiebedingte Morbidität ist die Unfruchtbarkeit [1]. Außerdem wird Frauen mit hormonsensiblen Krebsarten empfohlen, eine 5–10 Jahre andauernde adjuvante endokrine Therapie durchzuführen. Dadurch verschiebt sich das Eintreten einer möglichen Schwangerschaft und häufig führt dieser lange Zeitraum zu einem natürlichen Rückgang der Fruchtbarkeit [2].

Brustkrebs ist somit die häufigste Indikation für eine Fertilitätserhaltung („fertility preservation“ [FP]) bei Frauen im reproduktiven Alter [3]. Etablierte Methoden der FP sind die Kryokonservierung von Embryonen (in Deutschland nicht erlaubt), Eizellen und Eierstockgewebe [4]. Hierfür werden – wenn es der Zustand der Patientin erlaubt – kontrollierte Stimulationsbehandlungen („controlled ovarian stimulation“ [COS]) durchgeführt, um Embryonen oder Eizellen zu gewinnen. Diese Stimulationsbehandlungen führen im Körper der Frau zu überphysiologischen Östradiolspiegeln, die das Tumorwachstum bei hormonsensitiven BC theoretisch stimulieren könnten und zu einem Voranschreiten der Erkrankung und einer konsekutiv schlechteren Prognose führen könnten [5]. Mittlerweile wurden auch potenziell sicherere Stimulationsprotokolle mit Hinzunahme von Tamoxifen oder Letrozol entwickelt, um die Östrogenproduktion zu unterdrücken und das Risiko eines therapieinduzierten Tumorprogresses zu minimieren [6]. In früheren Berichten wurde auf die Notwendigkeit von größeren Studien zur onkologischen Sicherheit der FP hingewiesen. Das Ziel der vorliegenden Kohortenstudie war es, die langfristige Sicherheit der FP mit und ohne hormonelle Stimulation in einer großen, landesweiten Kohorte junger Frauen mit BC zu untersuchen.

## Patientinnen und Methoden

Es handelt sich um eine bevölkerungsbasierte, landesweite Kohortenstudie an Frauen im reproduktiven Alter (hier definiert als 18–44 Jahre), bei denen zwischen 1994 und 2017 in Schweden ein Brustkrebs diagnostiziert wurde. Für die zu untersuchende Kohorte wurden alle Frauen mit BC-Diagnose identifiziert, die sich zwischen dem 1. Januar 1994 und dem 30. Juni 2017 in einem FP-Programm der schwedischen Universitätskliniken einer FP unterzogen hatten. Die Kontrollkohorte (BC-Diagnose und keine FP) wurde anhand verschiedener Qualitätsregister ausgewählt [7]. Für jede Frau, die einer FP ausgesetzt war, wurden zwei Vergleichspersonen aus der Bevölkerung mit BC, aber ohne FP-Exposition nach dem Zufallsprinzip ausgewählt. Die Patientinnen wurden bezüglich der geografischen Region, des Alters und des Kalenderzeitraums bei der Diagnose abgeglichen. Ausschlusskriterien waren ein Carcinoma in situ, Fernmetastasen, Tumorstadium T4 und ein bilateraler Brustkrebs sowie Frauen ohne chirurgische Therapie. Insgesamt kamen 1275 Frauen für die Studie infrage, wobei das Verhältnis zwischen Kohorte und Kontrollkohorte bei 1 zu 2 lag. Durch die Verknüpfung mit mehreren schwedischen Bevölkerungsregistern konnten weitere Daten der Patientinnen abgerufen werden.

Die Variable war die FP, die nochmals in zwei Gruppen eingeteilt wurde: 1. Kryokonservierung von Eizellen und/oder Embryonen mit hormoneller Stimulation (hormonelle FP) und 2. Kryokonservierung von Eierstockgewebe ohne hormonelle Stimulation (nichthormonelle Stimulation). Eine Kombination aus beiden Verfahren wurde als hormonelle Stimulation kategorisiert. Die hormonelle FP wurde weiter nach der gleichzeitigen Verabreichung von Letrozol oder Standardstimulationsprotokollen unterteilt.

Der primäre Endpunkt war die krankheitsspezifische Sterblichkeit für die gesamte Kohorte. Der sekundäre Endpunkt war jedes Todesereignis oder das Auftreten eines Rezidivs. Daten zur Mortalität lagen für die gesamte Kohorte vor, wohingegen Daten zum Rezidiv nur für eine Subkohorte (723 Patientinnen) vorlagen. Das krebsspezifische Überleben wurde definiert als die Zeit bis zum Tod durch BC und das rückfallfreie Überleben als die Zeit bis zum Rückfall oder Tod.

## Ergebnisse

Die endgültige Studienpopulation umfasste 1275 Frauen, von denen sich 425 Frauen einer FP-Behandlung unterzogen: Kryokonservierung von Eierstockgewebe bei 58 Frauen, COS zur Kryokonservierung von Eizellen und/oder Embryonen bei 362 Frauen und eine Kombination dieser Methoden bei 5 Frauen. Das Rückfallrisiko wurde in der Subkohorte von 723 Frauen untersucht, von denen 198 mit hormoneller FP, 43 mit nichthormoneller FP und 482 ohne Exposition behandelt wurden.

Tod aufgrund von BC trat bei 17 Frauen mit hormoneller FP auf, bei 7 Frauen mit nichthormoneller FP und bei 80 Frauen ohne FP. Die mediane Nachbeobachtungszeit betrug 4,0 Jahre bei den Frauen mit hormoneller FP und 3,8 Jahre bei den entsprechenden Kontrollpersonen. Bei Frauen mit nichthormoneller FP und deren Kontrollpersonen betrug die Nachbeobachtungszeit 6,7 Jahre. In den statistischen Auswertungen ergab sich weder in der Kohorte „hormonelle FP“ noch in der Kohorte „nichthormonelle FP“ eine signifikant erhöhte Sterblichkeit im Vergleich zur Kontrollkohorte: Die bereinigte Hazard Ratio in Bezug auf die krankheitsspezifische Sterblichkeit lag bei Frauen mit hormoneller FP bei 0,59 und bei Frauen mit nichthormoneller FP bei 0,51.

Eine Subkohorte von 723 Frauen wurde weiter untersucht, um detaillierte Informationen über Rezidive zu beschreiben: Bei 97 Frauen ohne FP traten Rückfälle oder BC-bedingte Todesfälle auf. In der Gruppe „hormonelle FP“ waren es 26 Frauen und in der Gruppe „nichthormonelle FP“ waren es 9 Frauen. Die medianen Nachbeobachtungszeiten lagen bei 5,3 Jahren (Frauen mit hormoneller FP) und 4,9 Jahren in der Kontrollgruppe und bei 7,0 Jahren (Frauen mit nichthormoneller FP) und 6,9 Jahren in der Kontrollgruppe. Auch hier gab es keine statistisch signifikanten Unterschiede in der Rückfall- oder Sterberate zwischen den verschiedenen Gruppen. Die BC-spezifische Überlebensrate betrug in der Gruppe „hormonelle FP“ 96 %, sie lag bei 93 % in der Gruppe „nichthormonelle FP“ und bei 90 % in der Kontrollgruppe. Schätzungen des BC-spezifischen Überlebens nach 10 Jahren lagen bei 88 % in der Gruppe „hormonelle FP“, 90 % in der Gruppe „nichthormonelle FP“ und 81 % in der Kontrollgruppe. Die rezidivfreie 5‑Jahres-Überlebensrate betrug 89 % bei Frauen mit hormoneller FP, 83 % bei Frauen mit nichthormoneller FP und 82 % bei Frauen ohne FP. Nach 19 Jahren betrugen die Raten 82 %, 80 % und 73 %. Es gab im Vergleich COS mit oder ohne Letrozol keine signifikanten Unterschiede bei der BC-bezogenen Mortalität oder beim Rückfall.

## Schlussfolgerungen der Autoren

Die hier erhobenen Daten stehen im Einklang mit früheren Erkenntnissen [8]. Das krankheitsspezifische und das rezidivfreie Überleben bei Frauen mit BC-Diagnose und hormoneller FP und bei Frauen mit nichthormoneller FP ist genauso lang wie bei Frauen mit BC-Diagnose ohne FP-Behandlung. Die Ergebnisse liefern zusätzliche Erkenntnisse über die Sicherheit der FP-Verfahren und können im Praxisalltag Frauen mit BC-Diagnose, die ihre Fruchtbarkeit erhalten möchten, bei der Entscheidung helfen. Betroffene Patientinnen sollten bei Interesse an einer Fertilitätsberatung teilnehmen und die verfügbaren Informationen über die Sicherheit der angebotenen Verfahren erhalten.

## Kommentar

Diese landesweite schwedische Studie von Marklund et al. aus dem Jahr 2022 bringt weitere Sicherheit bezüglich des Themas Fertilitätserhalt bei gebärfähigen Frauen mit BC-Diagnose. Die große Fallzahl von 1275 Patientinnen erlaubt eine robuste Analyse und ergänzt die bisherigen Studien. Die Erhaltung der Fruchtbarkeit ist mittlerweile ein wichtiges Thema, das mit Patientinnen, bei denen Brustkrebs diagnostiziert wurde, besprochen werden sollte. Für Frauen wird die Kryokonservierung von Eizellen als erste FP-Option empfohlen, wenn genügend Zeit für die Durchführung einer COS bleibt. Bei Frauen mit ausreichender Ovarialreserve kann auch eine Kryokonservierung von Ovarialgewebe angestrebt werden, wenn eine Chemotherapie unverzüglich begonnen werden muss. Beide Verfahren sind in der hier durchgeführten Studie im Vergleich zu Frauen mit BC-Diagnose, die keine FP-Behandlung erhalten haben, als gleichwertig und gleich sicher anzusehen im Hinblick auf Überleben und Rezidivraten.

Ein Vorteil der Studie sind die große Größe der landesweiten Kohorte und ihre Vergleichspersonen, die mithilfe von bevölkerungsbezogenen Qualitätsregistern ausgewählt wurden. Den Registern konnten auch detaillierte Informationen zu Krankheits- und Behandlungsmerkmalen entnommen werden.

Obwohl die Studie eine längere Nachbeobachtungszeit aufweist als andere vorige Studien, wäre es wünschenswert, wenn die Nachbeobachtungszeit noch länger wäre. Ferner liefert die Studie keine Informationen zu anderen Outcome-Ereignissen wie zum Beispiel Therapiekomplikationen, Lebensqualität oder späteren Schwangerschaften. Es wäre wünschenswert, auch diese Faktoren zu untersuchen.

*Kathrin Hartwig, Kiel*
